# Anti-TGF-β Antibody Combined with Dendritic Cells Produce Antitumor Effects in Osteosarcoma

**DOI:** 10.1007/s11999-012-2299-2

**Published:** 2012-03-14

**Authors:** Masanori Kawano, Ichiro Itonaga, Tatsuya Iwasaki, Hiroyuki Tsuchiya, Hiroshi Tsumura

**Affiliations:** 1 Department of Orthopaedic Surgery, Faculty of Medicine, Oita University, Oita, 879-5593 Japan; 2 Department of Orthopaedic Surgery, Graduate School of Medical Science, Kanazawa University, Kanazawa, Japan

## Abstract

**Background:**

We previously reported the combination of tumor cryotreatment with dendritic cells to promote antitumor immunity. The effect of the combination treatment with dendritic cells and antitransforming growth factor-β (anti-TGF-β) antibody on the elimination of regulatory T cells and the inhibition of tumor growth was investigated.

**Questions/purposes:**

The effects of the combination treatment with dendritic cells and anti-TGF-β antibody on the enhancement of systemic immune responses and inhibition of metastatic tumor growth were investigated in a murine osteosarcoma (LM8) model.

**Materials and Methods:**

To evaluate activation of the immune response, we established the following three groups of C3H mice (60 mice total): (1) excision only; (2) tumor excision and administration of anti-TGF-β antibody; and (3) tumor excision and administration of dendritic cells exposed to cryotreated tumor lysates with anti-TGF-β antibody.

**Results:**

The mice that received dendritic cells exposed to cryotreated tumor lysates with anti-TGF-β antibody showed increased numbers of CD8(+) T lymphocytes, reduced regulatory T lymphocytes in the metastatic lesion, and inhibition of metastatic growth. The combined therapy group showed reduced numbers of regulatory T lymphocytes in the spleen and high serum interferon γ level.

**Conclusions:**

The control of the inhibitory condition induced by regulatory T cells is important to improve the suppression of the cytotoxic lymphocytes. Combining dendritic cells with anti-TGF-β antibody enhanced the systemic immune response.

**Clinical Relevance:**

We suggest that our immunotherapy could be developed further to improve the treatment of osteosarcoma.

## Introduction

Osteosarcoma has the highest frequency of occurrence of all malignant adolescent primary bone tumors [20]. The standard treatment consists of chemotherapy and surgical excision, and good results have been achieved in patients with osteosarcoma [8]. However, additional methods have yet to be developed for treatment of patients who are resistant to the standard osteosarcoma treatment [1]. Therefore, development of new treatment strategies for metastatic osteosarcoma is critical.

Nishida et al. [16] reported little beneficial antitumor effects after reimplantation of frozen tumor tissue alone, including ineffective inflammatory response and nonspecific reaction to tumor cells. To improve immunotherapy for osteosarcoma, we developed a method using dendritic cells (DCs) to enhance tumor-specific immunoreactions because DCs are the main antigen-presenting cells initiating cell-mediated immune responses in vivo [9].

To achieve stronger immune responses, it is important to control the immunosuppressive conditions in a tumor model. We focused on TGF-β which is important in regulation of the balance of immunity in nature [11]. TGF-β is one of the most important factors in a tumor model because it suppresses cell-mediated immunity by inducing regulatory T lymphocyte (Treg) production [4].

Tregs play an important role in maintaining the balance of immunity.

In the tumor progressive state, Tregs generally are activated and cell-mediated immunity is inhibited, particularly in DCs and cytotoxic T lymphocytes (CTLs) [4, 6, 11, 12, 24]. We therefore hypothesized that an antitumor effect might be provoked if Tregs are controlled, which would result in the activation of CTLs and inhibition of metastatic tumor growth.

The effect of combining dendritic cells and an anti-TGF-β antibody on enhancing the immune response to the tumor was examined.

Our study focused on (1) measuring the levels of Foxp3, a marker of regulatory T cells, and CD8 (+) T lymphocytes inside the metastatic tumor lesion to evaluate inhibition of the accumulation of regulatory T cells and the increase in cytotoxic T lymphocytes; (2) measuring changes in the metastatic tumor volume; (3) counting regulatory T cells using two markers, Foxp3 and CD4, in the spleen of mice to evaluate whether inhibition of regulatory T cells was attributable to treatment with the anti-TGF-β antibody; and (4) measuring the levels of IFN-γ as a marker of cell-mediated immunity and IL-10 as the suppressive factor of cell-mediated immunity.

## Materials and Methods

LM8 cells, derived from Dunn osteosarcoma, were provided by the Riken BioResource Center (Saitama, Japan). The cells were maintained in complete medium consisting of RPMI 1640 supplemented with 10% heat-inactivated fetal bovine serum, 100 μg/mL streptomycin, and 100 U/mL penicillin. Cells were cultured at 37°C in 5% CO_2_. A total of 1 × 10^6^ LM8 cells (a murine osteosarcoma cell line) was implanted hypodermically into the subcutaneous gluteal region of 60 female C3H mice that were 6 to 8 weeks old. We purchased these C3H mice from Sankyo Labo Service Corporation Inc (Toyama, Japan) and housed them in a specific pathogen-free animal facility in our laboratory.

**Fig. 1A–C:**
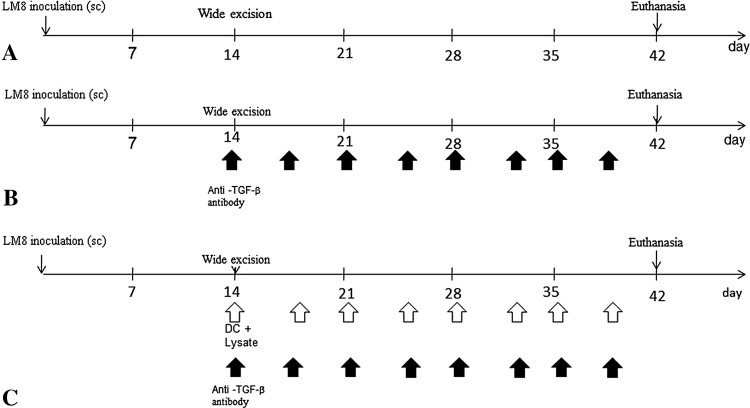
We established the following three groups: (**A**) EX, (**B**) EX + anti-TGF-β Ab, and (**C**) EX + DC(Ly) + anti-TGF-β Ab. SC = subcutaneous

All the animals had tumors develop. Three groups were established (Fig. 1): (1) the tumor was excised 14 days after inoculation (EX; n = 20); (2) the tumor was excised and intraperitoneal injection of anti-TGF-β antibody was performed twice per week (EX + anti-TGF-β Ab; n = 20); and (3) the tumor was excised and DCs exposed to cryotreated tumor lysates were injected twice a week into the subcutaneous contralateral gluteal region and intraperitoneal injection of anti-TGF-β antibody was performed twice per week (EX + DC[Ly] + anti-TGF-β Ab; n = 20). We did not observe any recurrence of the tumor at the primary site of inoculation after excision. All experiments were performed under the guidelines for animal experiments as stipulated by the Oita University Graduate School of Medical Science.

Bone marrow-derived DCs were generated as described by Lutz and Rössner [13] with minor modifications. Briefly, erythrocyte-depleted mouse bone marrow cells obtained from flushed marrow cavities (1 × 10^6^ cells/mL) were cultured in complete medium with 20 ng/mL recombinant mouse granulocyte macrophage colony-stimulating factor (GMCSF) (PeproTech EC Ltd, London, UK) in 10-cm tissue culture dishes at 37°C in an atmosphere containing 50 mL CO_2_ per L. The freeze-thawed tumor lysate was added to the DC cultures on Day 6 at a ratio of five DC equivalents to one tumor cell (ie, 5:1) and incubated at 37°C in an atmosphere containing 50 mL CO_2_ per L. After 24 hours of incubation, nonadherent cells including DCs were harvested by gentle pipetting.

After 2 weeks of tumor inoculation, the tumor mass was resected and soaked in liquid nitrogen for 20 minutes to kill the tumor cells. We added the freeze-thawed tumor lysate to the DC cultures on Day 6 at a ratio of five DC equivalents to one tumor cell (ie, 5:1). After 24 hours of incubation, nonadherent cells, including DCs, were harvested by gentle pipetting.

The monoclonal antibody 1D11 (mouse IgG1 neutralizes all three isoforms of TGF-β; 5 mg/kg) was provided by Genzyme Corporation (Framingham, MA, USA). The TGF-β-neutralizing activity of the 1D11 antibody was confirmed with the mink lung cell assay.

The markers Foxp3 and CD4, which are expressed on the surface of Tregs, were used to count the number of Tregs in the spleen by fluorescence-activated cell sorting (FACS) analysis. Foxp3 is a master gene for differentiation of Treg and it is a specific molecular marker of Tregs as well. CD4 is a superficial marker of helper T cells. Tregs is defined as a positive cell of Foxp3 and CD4 [22]. Tregs were counted with a FACS Calibur™ Flow Cytometer (Becton Dickinson, San Jose, CA, USA) and stained with fluorochrome-conjugated antibody (BD Pharmingen, Tokyo, Japan) for the following markers: Phycoerythrin (PE)-conjugated antimouse Foxp3 staining kit (eBioscience™, San Diego, CA, USA) and fluorescein isothiocyanate (FITC)-conjugated rat antimouse CD4 (BD Pharmingen™, clone: RM4-5). Data analysis was performed with CELLQuest™ software (Becton Dickinson).

Tumor volumes were measured using the micro-CT apparatus (R_mCT) which allows us to obtain high-resolution CT images in small living animals. The I-view-R (J. Morita Mfg Corp, Kyoto, Japan) was used as the viewer, and diagnosis was made with slice images viewed in all directions. Tumor volumes were estimated using the formula (π × long axis × short axis × short axis)/6.

IFN-γ is increased when the cell-mediated immunity is enhanced and IL-10 suppresses the cell-mediated immunity. We measured murine IFN-γ and IL-10 release by enzyme-linked immunosorbent assay using Quantikine^®^ (R&D Systems, Minneapolis, MN, USA) according to the manufacturer’s protocol using an Easy Reader EAR340 microtest plate reader (SLT Labinstruments, Salzburg, Austria).

Immunohistochemistry was used to measure the levels of Foxp3, a marker of Tregs, and CD8, a marker of cytotoxic T lymphocytes, inside metastatic tumor lesions. Lung specimens were fixed in 20% formalin and embedded in paraffin. In each case, we examined all formalin-fixed, paraffin-embedded tumor tissue blocks. Five samples per mouse were cut into 4-μm-thick slices. Rehydrated tissue sections were incubated with primary Abs against CD8 (+) (Santa Cruz Biotechnology, Santa Cruz, CA, USA) and Foxp3 (Abcam, Cambridge, MA, USA) diluted at 1:200 in Ab Diluent (Dako ChemMate, Kyoto, Japan) overnight at room temperature. For CD8 (+) staining with FITC donkey antirabbit IgG and Foxp3 (+) staining with Texas Red goat antirat IgG (Invitrogen, Carlsbad, CA, USA), secondary antibodies were diluted at 1:300 in Ab Diluent and added for 60 minutes at room temperature in the dark. Digital images were taken on a BIOREVO microscope equipped with a confocal microscopy system (BZ-9000; Keyence, Osaka, Japan).

We determined differences among the three groups using a nonrepeated measures ANOVA (and the Scheffe test). All analyses were conducted using SPSS^®^ 18.0 software (SPSS Japan Inc, Tokyo, Japan).

## Results

**Fig. 2A–E:**
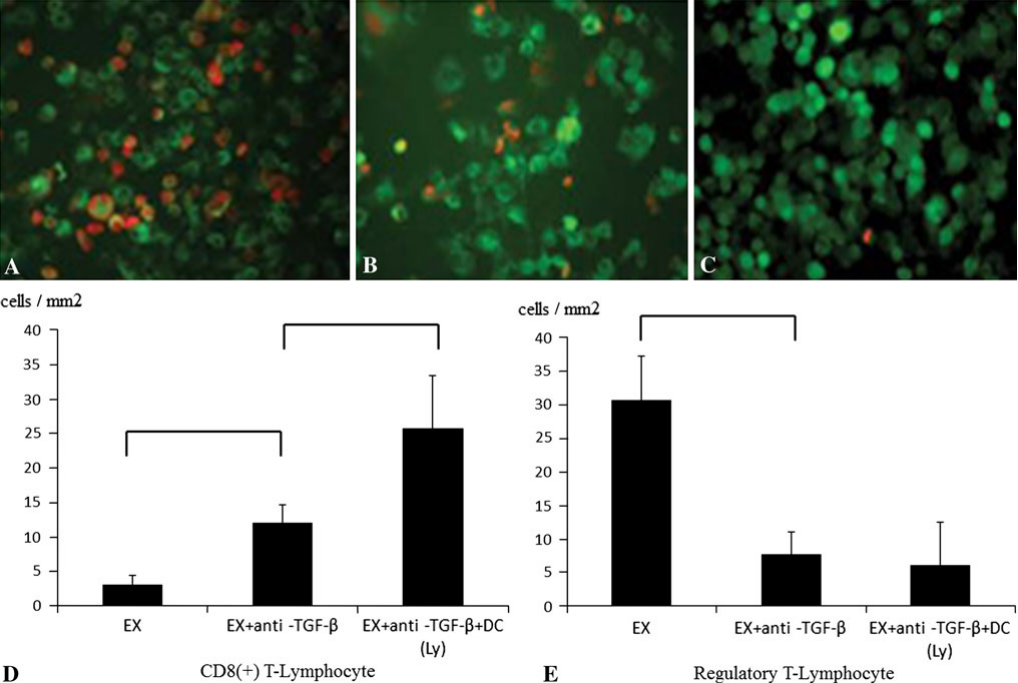
To evaluate CD8 (+) T lymphocytes and Tregs in pulmonary metastasis, immunostaining was performed in (**A**) the EX group, (**B**) EX + anti-TGF-β Ab group, and (**C**) EX + anti-TGF-β Ab + DC(Ly) group. The number of CD8 (+) T lymphocytes (Stain, FITC donkey anti-rabbit IgG; original magnification, ×200) was increased, and the Tregs (Stain, Texas Red goat anti-rat IgG; original magnification, ×200) were decreased in the combined therapy of the EX + DC(Ly) + anti-TGF-β Ab group. The numbers of (**D**) CD8(+) T lymphocytes and (**E**) Tregs per unit area in the three treatment groups are shown. Mice that received EX + DC(Ly) + anti-TGF-β Ab group showed the highest level of CD8 (+) T lymphocytes and the lowest level of Tregs. Error bars represent SD.

Foxp3, a marker of Tregs, was substantially decreased, and CD8 (+), a marker of cytotoxic T lymphocytes, was substantially increased in anti-TGF-β antibody-treated groups in metastatic tumor lesions. FOXP3 (+) cells were not recruited to the metastatic area in the anti-TGF-β antibody-treated groups compared with the excision-only group (Fig. 2A–C). The number of CD8(+) T lymphocytes per unit area was greater (p < 0.0001) in the mice that received DCs exposed to cryotreated tumor lysates with an intraperitoneal injection of anti-TGF-β antibody (25.73 ± 2.65 cells/mm^2^) than in the mice that received only an intraperitoneal injection of anti-TGF-β antibody (12.08 ± 7.61 cells/mm^2^) (Fig. 2D). The number of FOXP3 (+) T lymphocytes per unit area was lower (p < 0.0001) in the mice that received an intraperitoneal injection of anti-TGF-β antibody (7.73 ± 3.32 cells/mm^2^) than in the excision group (30.72 ± 6.61 cells/mm^2^) (Fig. 2E).

**Fig. 3:**
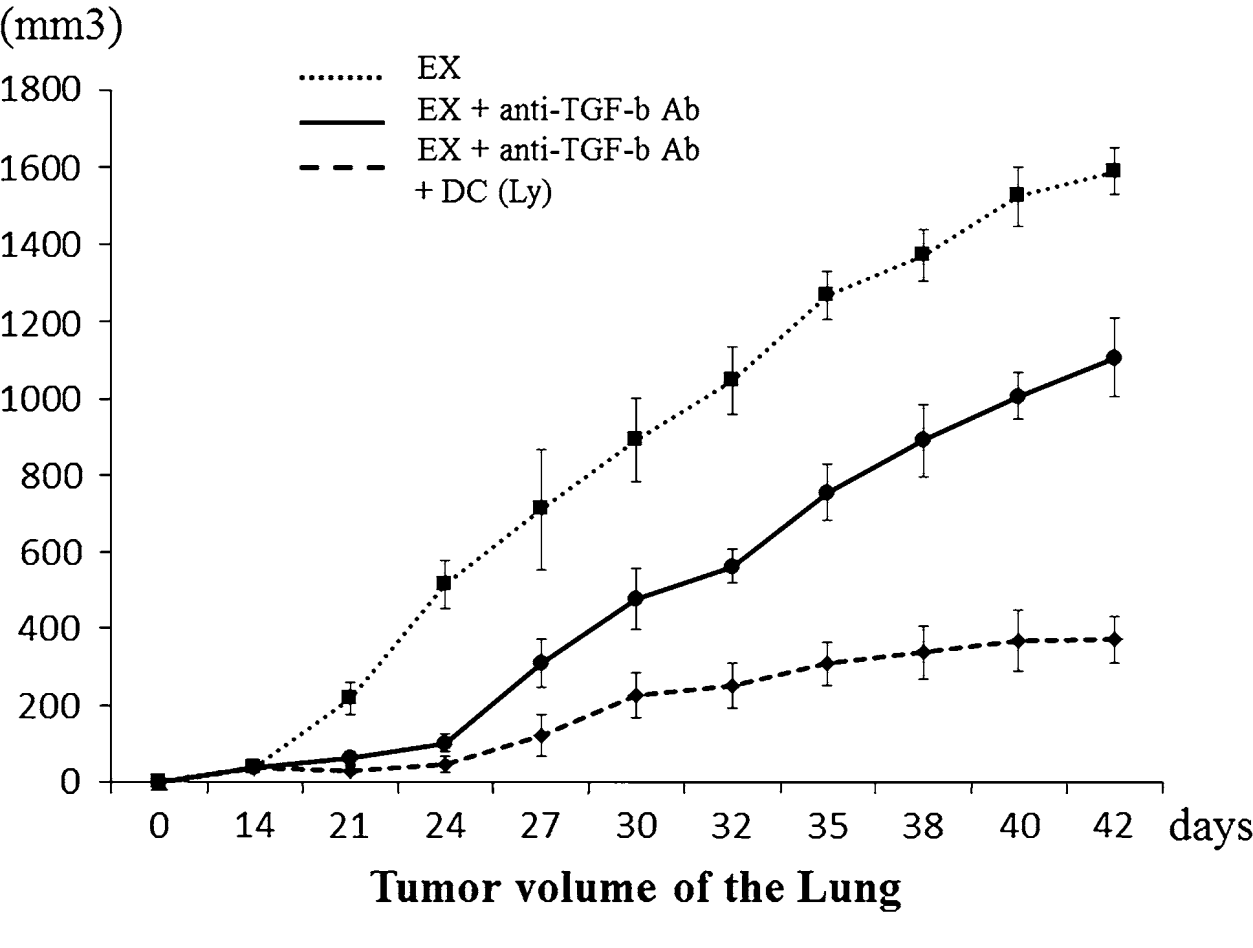
The volume of lung metastases in the three treatment groups is shown.

The change in metastatic tumor volume was smaller in the anti-TGF-β antibody and DC-treated group. The volume of the metastatic lesion (p < 0.0001) in the mice that received DCs exposed to cryotreated tumor lysates with intraperitoneal injection of anti-TGF-β antibody was significantly smaller (388.49 ± 34.48 mm^3^) than in the group that received only intraperitoneal injection of anti-TGF-β antibody (1104.53 ± 104.88 mm^3^) (Fig. 3).

**Fig. 4A–C:**
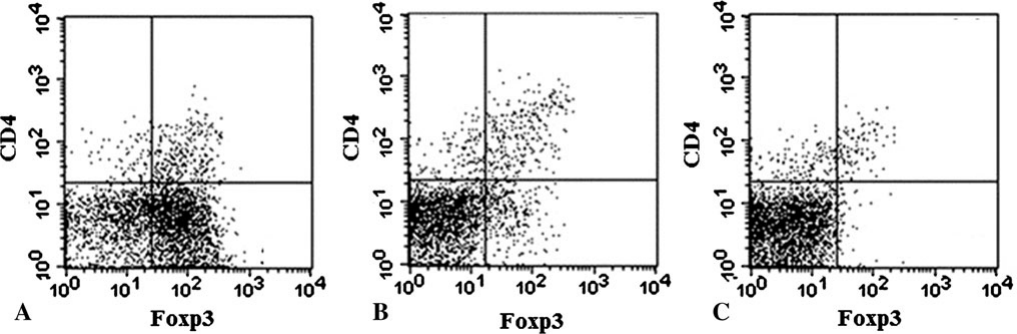
The ratio of CD4 (+) and Foxp3 (+)T cells in the spleen at Day 42 after tumor inoculation was measured using flow cytometry in (**A**) the EX group, (**B**) EX + anti-TGF-β Ab group, and (**C**) EX + anti-TGF-β Ab + DC(Ly) group. CD4 (+) Foxp3 (+) T cells decreased in the anti-TGF-β antibody groups.

The anti-TGF-β antibody was able to markedly reduce the cell population of the Tregs whose two markers are CD4 (+) and FOXP3 (+) in the spleen. The group that received an intraperitoneal injection of anti-TGF-β antibody showed a marked decrease in the percentage of CD4 (+) FOXP3 (+) cells compared with the excision group (Fig. 4).

**Fig. 5A–B:**
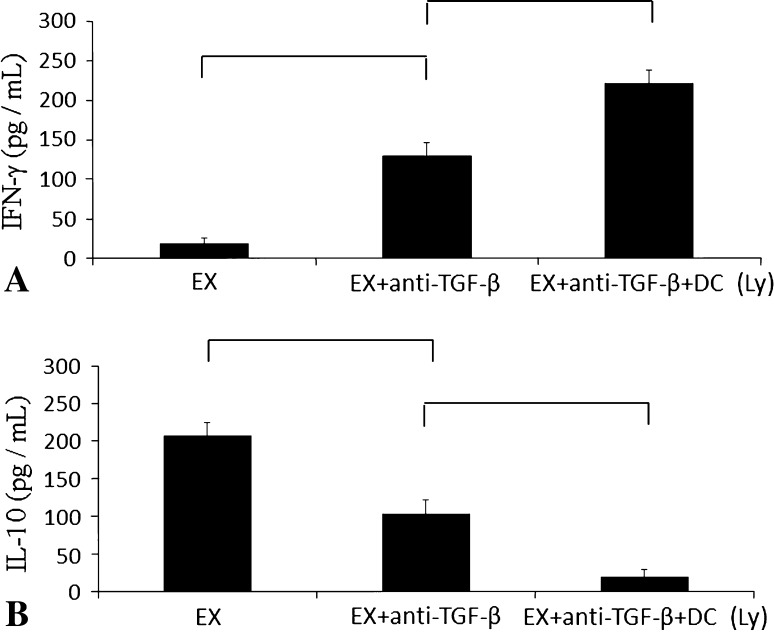
(**A**) Mice that received EX + anti-TGF-β Ab + DC(Ly) had the highest IFN-γ levels. (**B**) Mice that received EX + anti-TGF-β Ab + DC(Ly) had the lowest levels. Error bars represent SD.

An increase in serum IFN-γ level and a decrease in IL-10 level mean that cell-mediated immunity was enhanced in the DC and anti-TGF-β antibody-treated group. Mice treated with DCs exposed to cryotreated tumor lysates with intraperitoneal injection of anti-TGF-β antibody showed higher serum IFN-γ levels (221.81 ± 16.85 pg/mL) (p < 0.0001) than the group that received only intraperitoneal injection of anti-TGF-β antibody (130.01 ± 16.07 pg/mL). Serum IL-10 was lower (p < 0.0001) in the mice that received DCs exposed to cryotreated tumor lysates with intraperitoneal injection of anti-TGF-β antibody (20.33 ± 9.05 pg/mL) than in the group that received only intraperitoneal injection of anti-TGF-β antibody (103.73 ± 18.37 pg/mL) (Fig. 5).

## Discussion

Although adjuvant therapy is the most effective treatment strategy for osteosarcoma, new treatment methods for metastatic osteosarcoma should be developed. In the 1970s, immunotherapy for osteosarcoma was reported by Marcove et al. [14], Southam et al. [19], Marsh et al. [15], and Gainor et al. [3]. In addition, several immunotherapies have been attempted as new methods to overcome progressive cancer [7, 17, 18] (Table 1). We focused on the inability to control the factors that inhibit the attacker cells such as DCs and CD8(+) T-lymphocytes as a major cause of insufficient antitumor effects. TGF-β plays an important role by inducing Tregs [12]. Our purposes were to (1) evaluate the levels of Foxp3and CD8 (+) T lymphocytes inside the metastatic tumor lesion; (2) determine the changes in metastatic tumor volume; (3) count the regulatory T cells in the spleen of mice to evaluate inhibition of regulatory T cells; and (4) measure the levels of IFN-γ and IL-10.

**Table 1:** Immunotherapeutic trials in malignant tumors

Study	Tumor	Immune intervention	Route	Immunologic response	Comments
Marcove et al. [14]	Osteogenic sarcoma	Irradiated autogenous vaccine	SC	NC, PD	Activate immunization using homogenized tumor cell is encouraging
Southam et al. [19]	Osteogenic sarcoma	Irradiated autogenous vaccine	SC	NC, DOD	The relatively high proportion of patients who apparently are cured
Marsh et al. [15]	Osteosarcoma	Lymphocyte sensitization	IV	NC, DOD	Sensitized lymphocytes may favorably influence the course of osteosarcoma
Gainor et al. [3]	Osteosarcoma	Lymphocyte proliferation assay	In vitro	In vitro	LPA may be useful in monitoring clinical progress of the disease
Soleimani et al. [18]	Renal cell carcinoma	DCs and tumor lysate	SC	NC, PD	Depletion of regulatory T cells should be attained by combination of conventional cancer treatment and cancer vaccine immunotherapy
Rosenberg et al. [17]	Melanoma	T lymphocyte	IV	PR, CR	TILs can mediate durable complete responses
Jähnisch et al. [7]	Prostate cancer	DCs	SC	NC, PD	DCs can directly mediate tumor-directed cytotoxicity
Current study	Osteosarcoma	DCs and TGF-β	SC, IP	PR	Combining DCs with anti-TGF-β antibody resulted in enhanced antitumor effects

DCs = dendritic cells; SC = subcutaneous; NC = no change; DOD = dead of disease; PR = partial response; IV = intravenous; IP = intraperitoneal; CR = complete response; PD = progressive disease; LPA = lymphocyte proliferation assay; TILs = tumor-infiltrating lymphocytes.

There were some limitations to this study. First, we used mice with an identical genetic makeup and DCs from a different (albeit genetically identical) mouse in transfer experiments. Clinical applications would require the use of DCs derived from the same individual, which was not possible in our mouse model. Second, because we used mouse osteosarcoma cells and DCs, we were unable to test the induction of human osteosarcoma and the data may not reflect the responses of the human immune system. Further studies are required to confirm these observations across a wide range of models for clinical application.

Anti-TGF-β antibody inhibited the accumulation of Tregs and induced the infiltration of CD8 T cells inside the metastatic lesion. TGF-β signaling in CD4 (+) CD25(+) Treg is required for their immunosuppressive capacity [6]. Hawinkels et al. [5] reported TGF-β levels were significantly increased in tumor tissue compared with adjacent normal tissues. We showed blockage of TGF-β led to the inhibition of Foxp3 (+) T cells inside the tumor tissues. The inhibition of Tregs therefore produced desirable results in our study. However, the inhibition of TGF-β and Tregs enhances antitumor immunity and the risk of autoimmune diseases developing or cytokine storm. Establishing the adequate dosage of antibody and careful monitoring to prevent the occurrence of severe adverse effects are necessary for clinical application of this antitumor therapy.

The group treated with the combination of DCs and anti-TGF-β antibody also showed suppression of the size of lung metastases. Tregs are a major component of the immunosuppressive microenvironment of metastatic lesions [24]. This is consistent with our results that metastatic lesions were substantially inhibited in the combined therapy group, suggesting the control of the immunosuppressor factors may facilitate the activity of CTLs in the tumor microenvironment.

Anti-TGF antibody inhibited the proliferation of Tregs in the spleen. Inhibition of Tregs accumulation in the spleen can produce enhancement of the systemic cell-mediated immunity through activation of the DCs or cytotoxic T lymphocytes. We believe that this could suggest the reduction of Tregs and proliferation of CD8 (+) T lymphocytes inside lung metastatic lesions (Fig. 2).

Blockage of TGF-β induced activation of cell-mediated immunity by increasing serum IFN and reducing IL-10. TGF-β causes potent immunosuppression mediated by cytokines from tumor cells, and TGF-β blockage may be useful for enhancing cancer therapy or vaccine efficacy [10, 21, 23, 25]. Our results for the serum cytokine levels suggest that the anti-TGF-β antibody not only inhibited the accumulation of regulatory T lymphocytes as immunosuppressors but also enhanced cell-mediated immunity.

Currently, chemotherapy for human osteosarcoma is the standard treatment, and it should be administered before immunotherapy. The effectiveness of the anti-TGF-β antibody should be evaluated in combination with current chemotherapies for treatment of human osteosarcoma. Biswas et al. reported that mice treated with the chemotherapy drug doxorubicin showed elevated levels of TGE-β and tumor spread [2]. Therefore, the combination of chemotherapy with anti-TGF-β antibody could be effective for treatment of patients with residual tumors or distant metastasis after chemotherapy. These issues will be addressed in future studies to enable clinical application of our therapy for treatment of human osteosarcoma and to facilitate further research efforts.
